# Activated learning; providing structure in global health education at the David Geffen School of Medicine at the University of California, Los Angeles (UCLA)– a pilot study

**DOI:** 10.1186/s12909-016-0581-9

**Published:** 2016-02-16

**Authors:** Jaime Jordan, Risa Hoffman, Gitanjli Arora, Wendy Coates

**Affiliations:** David Geffen School of Medicine, University of California Los Angeles, 10833 Le Conte Ave, Los Angeles, CA 90095 USA; Department of Emergency Medicine, Harbor-UCLA Medical Center, 1000 W Carson St, Torrance, CA 90502 USA; Los Angeles Biomedical Research Institute at Harbor-UCLA, 1124 W Carson St, Torrance, CA 90502 USA

**Keywords:** Global health education, Undergraduate medical education, Global health, Medical student, Curriculum development

## Abstract

**Background:**

Global health rotations are increasingly popular amongst medical students. The training abroad is highly variable and there is a recognized need for global health curriculum development. We sought to create and evaluate a curriculum, applicable to any global health rotation, that requires students to take an active role in their education and promotes engagement.

**Methods:**

Prospective, observational, mixed method study of 4th year medical students enrolled in global health courses at UCLA in 2011–12. Course directors identified 4 topics common to all rotations (traditional medicine, health systems, limited resources, pathology) and developed activities for students to complete abroad: observation, interview and reflection on resources, pathology, medical practices; and compare/contrast their experience with the US healthcare system. Students posted responses on a discussion board moderated by US faculty. After the rotation, students completed an anonymous internet-based evaluative survey. Responses were tabulated. Qualitative data from discussion board postings and free response survey items were analyzed using the framework method.

**Results:**

14 (100 %) students completed the Activated Learning assignment. 12 submitted the post rotation survey (85.7 %). Activated Learning enhanced GH education for 67 % and facilitated engagement in the local medical culture for 67 %. Qualitative analysis of discussion board posting demonstrated multiple areas of knowledge gain and analysis of free response survey items revealed 5 major themes supporting Activated Learning: guided learning, stimulation of discussion, shared interactions, cultural understanding, and knowledge of global healthcare systems. Increased interactivity emerged as the major theme for future improvement.

**Conclusion:**

The results of this study suggest that an Activated Learning program may enhance education, standardize curricular objectives across multiple sites and promote engagement in local medical culture, pathology and delivery systems. Increased interaction between students and faculty may augment the impact of such a program.

## Background

Global health is the study, research and practice of improving health and achieving equity in health for all people worldwide through international and interdisciplinary collaboration [1]. This discipline is gaining interest amongst medical trainees and a greater number of students are enrolling in global health experiences [2, 3] to explore different cultures, regional pathology, and healthcare delivery systems. In order to meet this demand, educators have begun to develop and implement curricula focusing on global health topics and providing elective rotations for learners abroad. Likewise, there has been rapid expansion of organizations dedicated to global health education, such as the Global Health Education Consortium (GHEC), American Medical Association Office of International Medicine, and the Global Health Action Committee of the American Medical Student Association [4].

Despite these recognized first steps to accommodate educational demand, there is no standardization in global health education for medical students [3] and some educators question the value of international experiences [5]. There is tremendous variability in the relationship between the home school and the international site with regard to student roles, involvement, and supervision as well as different needs, expectations, structure, and motivations for these educational collaborations. In some global health education models, US based medical schools take the lead in arranging global health rotations with international institutions and may send US based faculty to accompany the students. Other programs may be in close contact with faculty at the international site and pre-arrange learning goals and objectives for visiting students. Additionally, US schools may arrange for its students to rotate in a global health setting where they are integrated into the international site’s own goals and objectives. Finally, students may arrange their own global health experiences and seek approval from their home institution to secure credit for the time spent. With such variability, student experience may be vastly different, with respect to clinical responsibility and experience and involvement of local providers in supervisory and teaching roles. The clinical settings range from an established medical facility, a periodic mobile clinic to a remote site, or a rescue mission commissioned by a US based organization. Each of these models has the potential for a rich learning experience. While prior literature has suggested positive educational benefits to global health and international medicine rotations [6–8], a lack of standardized goals and learning objectives can lead to a suboptimal learning experience. There is a recognized need for curriculum development in global health education [9]. Global health curricular frameworks and core content have been proposed [4, 10–13], but there is still a lack of consensus as to best practices or appropriate training in global health for medical trainees [14].

### The University of California Los Angeles Global Health Education Program

To meet the increased demand for global health experiences and to ensure a high quality educational experience, in 2010 the David Geffen School of Medicine at the University of California, Los Angeles (UCLA) established the Global Health Education Program (GHEP). A signature GHEP component is the Global Clinical Elective, a month-long rotation at a UCLA partner site for fourth year medical students. Current partner sites are located in South Africa, China, Mozambique, Malawi, Thailand, and Peru and range in focus from primary care to infectious disease to emergency medicine. Rotations are available to UCLA medical students through a competitive application process. Funding is provided to students accepted in the program and students are required to attend a comprehensive in-person pre-departure orientation, agree to a code of conduct, and complete pre-departure learning assignments designed for self-learning on clinical topics relevant to the partner site. GHEP also requires students to debrief about their experiences with GHEP faculty on return.

During the 2011–2012 academic year, GHEP sought to provide additional mentorship and learning during the students’ global health rotation. Although students were receiving pre-departure training, teaching from partner site faculty, and post-return debriefing, there was a recognized opportunity for GHEP faculty familiar with the partner sites to provide mentored learning on specific topics during the rotation that spanned all sites. The curriculum, entitled ‘Activated Learning’ was created as an educational innovation to encourage students to take an active role in their education during their global health elective by participating in specific activities designed to promote engagement with the local medical community, healthcare systems, and their colleagues. The objective of this study was to provide structure to existing global health curriculum and direct student education across multiple international sites to meet pre-established and standardized learning outcomes.

## Methods

### Study setting and participants

Fourth-year medical students enrolled in global health clinical electives as part of the 2011–2012 Global Health Education Program were eligible to participate in the Activated Learning curriculum and evaluation. The sites offered unique experiences for students to meet individual preferences; Malawi (infectious diseases), Mozambique (pediatrics), South Africa (emergency medicine and pediatrics), China (primary care and medicine subspecialties), Peru (primary care and infectious disease), and Cameroon (general surgery). This study was certified as exempt by the Institutional Review Board from the David Geffen School of Medicine at UCLA Office for Protection of Human Subjects.

### Study design

**Fig. 1:**
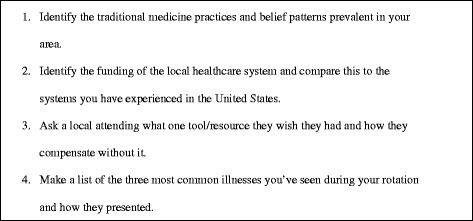
“Activated learning list” student assignment

This was a prospective, observational, mixed methods study. Prior to curriculum development, key course faculty at the home institution performed a literature review on existing curricula and through in depth discussion and consensus identified four major topics felt to be of high importance and common to all our global health rotations – traditional medicine, healthcare systems, resource limitations, and region specific disease pathology. These topics are consistent with the core competencies suggested by the Association of Faculties of Medicine of Canada Resource Group/GHEC committee [15]. Learning activities were created by a group of experienced educators to address these key topics and consisted of observations, interviews, and compare/contrast healthcare systems (US vs. global health site). This was called the “Activated Learning List” (Fig. 1). At the pre-departure orientation, the Activated Learning Assignment was described. Students were informed that these topics were designed to further their learning of the health system and the country and culture they would be visiting and that they would be assigned a weekly activity that would require the posting of a reflective response on an online discussion board in real time. These posts would be viewed by fellow students on Global Clinical Electives and moderated by GHEP faculty based at UCLA. Students were encouraged to share their own experiences and comment on the experiences of their colleagues. Faculty provided comments and feedback to students via the discussion board to share experiences, clarify concepts and ask further thought provoking questions. The goal of the discussion board was to create a learning community among students that spanned all sites abroad as well as the global health faculty at the home institution, where knowledge could be shared. Guidelines for posting on the blog were discussed during the pre-departure orientation, including that the blog was password protected, the content to be shared only with fellow GHEP Clinical Elective students and GHEP faculty and staff, that for those participating in the study shared comments would be made anonymous for research purposes, and that experiences should be discussed in a professional manner. Once the students returned to Los Angeles, they completed an evaluative survey of the Activated Learning assignment, consisting of Likert Scale and free response items.

### Data analysis

Qualitative analysis was performed using the framework approach [16–18] with identification and coding of themes for discussion board postings and the post-rotation free response survey items. Coding was verified by agreement by two independent reviewers (JJ and WC). Both reviewers had assisted in the design of the Activated Learning List and were familiar with the goals of the exercise. Discrepancies were resolved by in-depth discussion and negotiated consensus. Descriptive statistics were reported for post-rotation Likert Scale survey items.

## Results

All 14 students enrolled in global health electives during the study period participated in the “Activated Learning list” activities and discussion board. Twelve students completed the post rotation evaluative survey for a response rate of 85.7 %. Major themes identified in the discussion board postings are presented in Table 1. Students were able to learn important concepts regarding cultural beliefs and traditional medicine, financial aspects of local healthcare systems and inequities of care, the limitations of resources in various environments and how these limitations affect expectations of healthcare outcomes, and common disease presentations (Table 1).

**Table 1:** Discussion board results

Question	Major themes	Number of comments	Example
Identify the traditional medicine practices and belief patterns prevalent in your area.	Primary role of traditional medicine	9	“The traditional medicine, or ‘country medicine’ was a force to be reckoned with in Cameroon.”
	Prominent societal role of traditional practitioner	6	“Herbalists in Malawi are licensed to practice by the Malawian government. He/she fills a great position in society.”
	Symbolism	8	“We have been seeing woven bands and strings tied around kids’ waist or wrist. When we asked about the mothers about the significance, they had difficulty putting it into words but said it offered ‘protection’.”
	Physical intervention	5	“Many kids show up with multiple 1-2 cm vertical scars on their abdomens and we’ve heard various explanations for them.”
	Herbs/medicine	5	“We rotated through a traditional Chinese medicine pharmacy, which encompassed a highly diverse portfolio of herbs, roots, animal products, and minerals. The most surprising included mammalian fossils, centipedes, burned human hair, water buffalo horn shavings, and armadillo scales.”
	Allopathic medicine as rescue therapy	9	“More often than not, and maybe out of convenience and location, patients would often visit their local healers first. Their visit to the hospital often came only after traditional medicine failed.”
	Complications of traditional medicine practices	7	“During my four-week rotation, there were at least 4 children who presented with signs of ileus and underwent exploratory laparotomies only to find that they had been given a green leaf herb that was an extremely potent anticholinergic agent.”
Identify the funding of the local healthcare system and compare this to the systems you have experienced in the United States.	Patient contribution/fee-for-service	7	“The patient was required to pay the estimated cost partially or in full prior to their admission and if they didn’t pay, they wouldn’t receive the required operation.”
	Government sponsored	8	“The majority of services provided here are funded by the health services arm of the federal government.”
	External aid	5	“Much of Mozambican Ministry of Health is financed by foreign aid and mandated philanthropy from foreign enterprise operating within Mozambique.”
	Physician compensation	4	“Most physicians are compelled to fulfill their obligation to [the government] by staffing the public hospital on weekday mornings and then working at private hospitals and clinics in the afternoons to supplement their incomes.”
	Inequities of care	10	“Government spending on health is split between the public and private sectors with 50 % going to the public and 50 % going to the private sectors. However, the private sector only serves 15 % of the population while the public sector serves 85 %.”
Ask a local attending what one tool/resource they wish they had and how they compensate without it.	Training	6	“Physicians practicing in [rural] areas often have only high school vocational training, rather than five or seven years of medical school common in larger cities.”
	Personnel	4	“…what was in shortest supply was strongly trained nurses.”
	Technology/equipment	6	“There were no ventilators, defibrillators, a-lines, monitors, etc.”
	Lack of basic supplies	5	“Consistent electricity was a serious problem that always needed to be considered.”
	Improvisation	6	“For instance, [there is a lack of spacers for inhalers], so plastic bottles that were used to hold saline were cut up so that they could fit in the inhaler.”
	Importance of the physical exam	4	“The amazing outcome that arose as a result of the absence of these modalities was how much the physical exam meant when a decision needed to be made…”
	Different expectations of healthcare outcomes	5	“Every patient carried a virtual DNR label because there were simply no measures to keep a resuscitated patient alive if it was in fact successful.”
Make a list of the three most common illnesses you’ve seen during your rotation and how they presented.	Infectious disease	9	“Tuberculosis and related complications – pneumonia, meningitis.”
	HIV	7	“There are some children as young as one-or two years old who, at such a young age, are already on second line antiretroviral therapy because their caretakers had been non-adherent with the regimen and resistance developed.”
	Gastrointestinal disease	7	“Diarrhea was perhaps the most common reason for visits to the clinic.”
	Signs and symptoms	14	“The most common presentation for adults has been productive cough and weight loss but there is great variability in presentation due to the high prevalence of HIV and AIDS in the community.”

In regard to health systems issues, students not only identified facts regarding resources and logistics, but also demonstrated an understanding of how these facts impact individuals and public health. As one student aptly put:

“In order to be seen at a private hospital, patients must put down a 5,000 Rand deposit (658 USD) in order to be seen by a doctor. The average income for employed persons is 5000–10000 Rands per month. This effectively prevents poorer patients from being able to access care in the private sector.”

Another student commented:

- “Every patient carried a virtual DNR [Do Not Resuscitate] label because there were simply no measures to keep a resuscitated patient alive if it was in fact successful.”

Students also demonstrated an in-depth exploration of cultural practices and their complex relationship with health. Some exemplary comments include:

- “The traditional medicine, or ‘country medicine’ was a force to be reckoned with ……There are very few restrictions regarding who can and cannot practice medicine. Virtually anyone who had any form of medical education, or claimed to have been educated, could open a practice and treat patients with any form of medicine. Nearly every patient had some form of physical evidence that the local healers had treated them at one time in their lives. On two occasions men with hydroceles came to the hospital after they were aspirated with used needles or incised with heated blades. The outcome in both cases was fournier’s gangrene.”
- “A less common practice among a subset of [local] families is to cut off the tip of the 4th finger, down to the DIP joint. One mom explained that it was frequently done either for protection or to cure an ailment. The example she gave was that her sister had been wetting her bed, and after they cut her finger, the bed-wetting stopped. One of the attendings we talked to mentioned that although this practice is part of their cultural beliefs, it also does lead to the intended result given the severe negative reinforcement.”

**Fig. 2:**
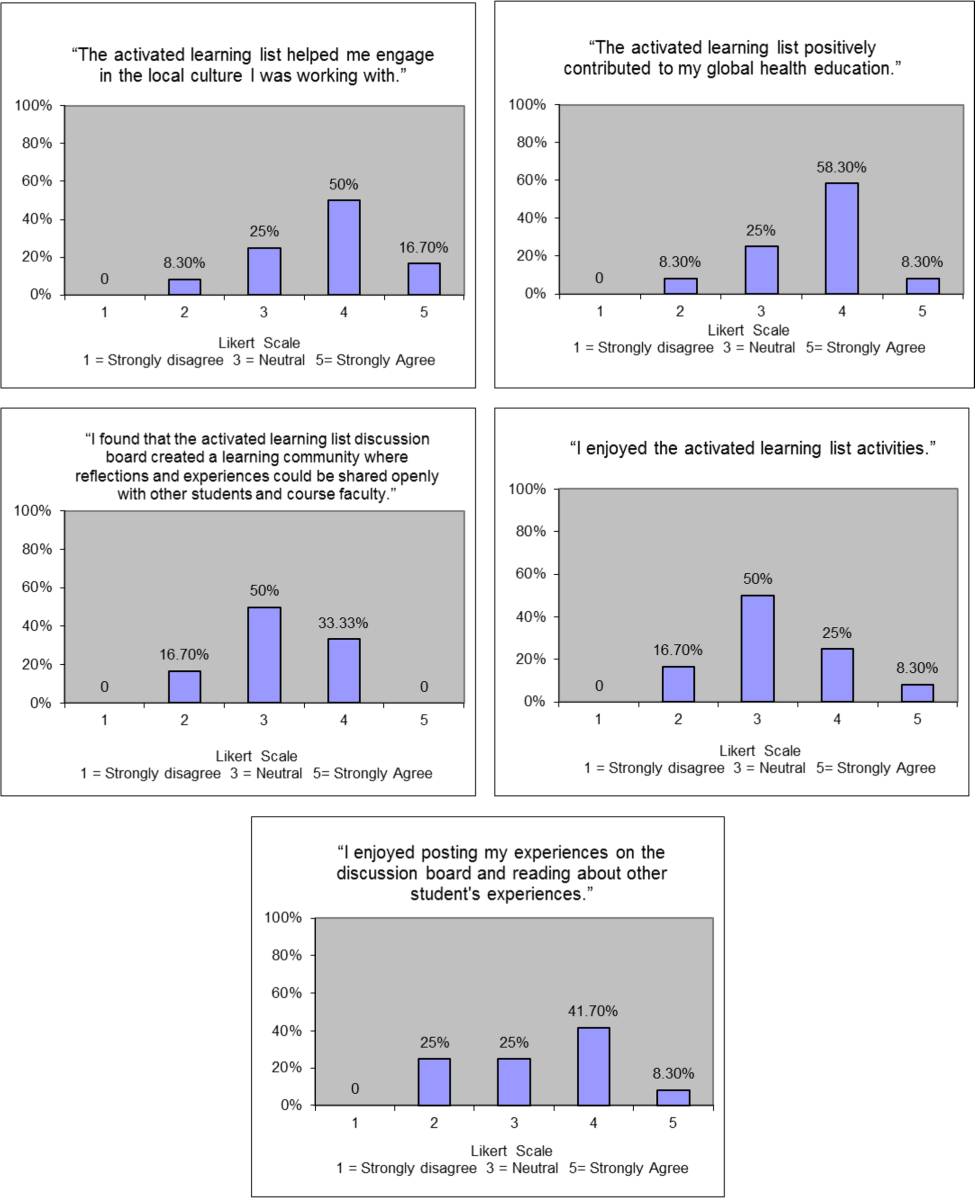
Survey results (likert scale items)

Survey responses for Likert Scale items are displayed in Fig. 2. Overall 67 % of students felt that the Activated Learning list positively contributed to their global health education and 67 % felt that this program promoted engagement with the local community and culture. Approximately 33 % of students felt that the discussion board created a learning community and 50 % were neutral. Qualitative analysis of the free text items revealed 5 major themes on how the program positively contributed to student global health education: guided learning, stimulation of discussion, encouragement of interactivity, cultural understanding, and knowledge of healthcare systems (Table 2). One major theme that emerged for future improvement in the Activated Learning activity was the need for increased interactivity (Table 2).

**Table 2:** Survey results (free response items)

Question	Major themes	Number of comments	Example
List 3 ways the “Activated Learning List” experience contributed to your education.	Guided Learning	7	“It got me to think outside the box.”
	Stimulated Discussion	3	“The questions inspired several great discussions with the people we worked with at the hospital.”
	Encouraged Interactivity	8	“… comparison with other countries, and collaboration with the students in my country.”
	Cultural Understanding	6	“It made me think about important cultural issues I had not considered.”
	Knowledge of Healthcare Systems	5	“It helped me to understand how the health system is structured.”
What “Activated Learning” activities would you suggest for next year?	Culture and Customs	2	“Similar questions regarding local cultural beliefs relevant to health problems and funding of care delivery.”
	Healthcare systems	7	“I would be interested in learning about how medical delivery personnel are trained in each place.”
	Increased Interactivity	6	“Video blog”
How can the “Activated Learning List” experience be improved for future students?	Increased Interactivity	6	“…Create an improved forum for discussion to allow for more back and forth conversation.”

## Discussion

The results of this pilot study suggest that Activated Learning contributes positively to medical students’ global health experiences, and we believe this model has the potential to enhance learning. With the increasing demand for education in global health by medical trainees, it is essential to develop and evaluate curricula in global health with the same rigor as for other medical school program components. We felt that “activating” students would help guide and deepen their learning. We sought to provide some structure by directing student learning, in a way that was not too rigid to detract from the pure experience of being abroad and participating in another healthcare environment. From analyzing the discussion board postings, it is clear that students are gaining a great deal of knowledge and understanding of the healthcare environment and culture that they are working in with this curriculum.

While the literature describes some similar experiences of students who are engaged in global health experiences, it is not known whether this is a guaranteed result. It is possible that insightful and experienced students may indeed ask similar questions and are then likely to report their findings in the literature. However, some students may be uncomfortable with asking direct questions about health access, cultural issues, or clinical concerns and others may not even realize the significance of knowing these answers as a basis for a better overall experience. We sought to empower our students in advance. Our assignment also laid the groundwork to foster relationships with providers at all levels to give students a broader perspective of the state of health care delivery at their sites.

We noted a conflict in the evaluative survey between the numeric ratings on our Likert scale items and the free text comments of the learners, with lower numeric ratings but highly favorable written comments. As the program matures, it will be important to gauge learner satisfaction with the Activated Learning curriculum with more detailed evaluations to better understand student perspectives. It will also be important to identify potential barriers to student acceptance of Activated Learning, and to modify the curriculum to suit the needs of the learners through continuous review and content modification, including an analysis of the faculty responses and contributions to the discussion board.

The main theme that emerged from the evaluation for improvement in Activated Learning was the need for increased interactivity, which is not surprising as a high level of interactivity is important factor in the success of an educational program [19–21]. This is particularly challenging in global health rotations when faculty and learners are separated by time and space. We attempted to create meaningful interaction through the design of our activities and through inclusion of a discussion board in attempts to create a “community of inquiry”, which has been previously shown to have a profound impact on learning outcomes and satisfaction in asynchronous education [20, 22]. Creating improved formats and channels for communication and back and forth discussion will be key in the success of Activated Learning.

The major goal of this program was to provide structured educational objectives to “prime” students, making them aware of potential areas of learning to complement the current curriculum and deepen their experience while abroad. It also provided students with a forum to connect with familiar people and glean insight from colleagues who faced similar issues in nearly real-time as well as reliable access to faculty at the home institution if needed as a support mechanism. The results of this pilot study suggest that we were able to accomplish these goals, but identified the need for improved interactivity while abroad. Based on student feedback, modifications for upcoming years include a revised list of Activated Learning activities and an improved discussion board format to allow for more back and forth conversation among both the students and the home-based faculty.

### Limitations

This is a pilot study, and while study activities were conducted at multiple global health sites, all participants came from a single institution, so the results may be difficult to generalize. It will be important in the future to perform larger studies at multiple institutions. A future analysis should address higher-level outcome measures, including demonstrated knowledge acquisition and behavioral change in clinical decision-making [23]. Potential strategies for accomplishing this would be an analysis of preceptor evaluations, formal knowledge testing, or structured interviews upon return. For this study, we kept the evaluation short to maximize the number of responses. In the future, we may wish to seek detailed information on all domains of interest, such as more in depth reasons for ratings, impact on stress level of the student (i.e. was it an added burden and how much extra time did it take) and also solicit feedback from site preceptors to learn their views on how Activated Learning may impact their experience with students. In future studies, it would also be important to question the students about the negative aspects of their experience abroad in order to paint a more complete picture of the overall experience.

## Conclusion

The Activated Learning program strengthened our global health rotations by providing a standardized curriculum and promoting engagement with local medical culture, regional specific pathology and healthcare delivery systems. It also provided an opportunity for students to engage in a familiar community while separated from their home institutions. Students met the specified learning outcomes. Activated Learning is simple, easy to implement and can be incorporated into the curriculum with little to no cost. Each institution can tailor an Activated Learning program to fit with their master curricular plan. Increased interaction between students at different sites and US-based faculty may augment the impact of Activated Learning. The results of our study suggest that Activated Learning can be a beneficial tool for global health programs. Prospective comparative studies with a larger sample size are needed to more rigorously evaluate the benefit to students and impact on host institutions.
